# Supplementary material for: Engineering stringent genetic biocontainment of yeast with a protein stability switch
Source: Nat Commun. 2024 Feb 5;15:1060. doi: 10.1038/s41467-024-44988-8 (PMC10844650; doi:10.1038/s41467-024-44988-8)
Supplement: Supplementary file 1 — Supplementary Information [file 41467_2024_44988_MOESM1_ESM.pdf]

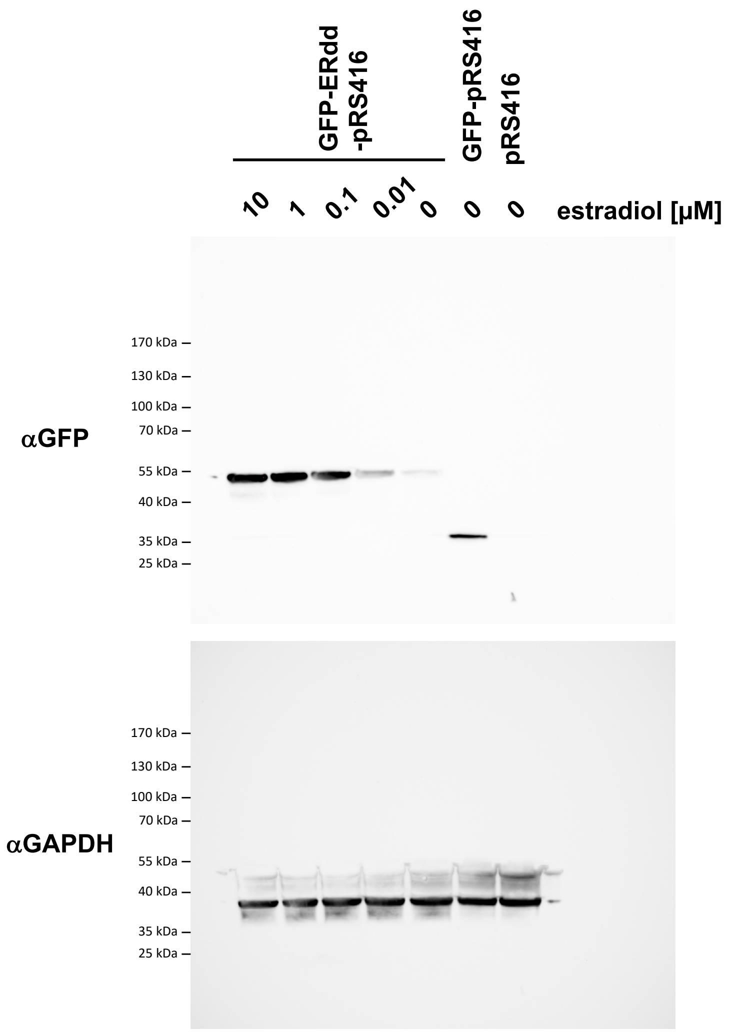

Figure S1: Uncropped western blot of yeast lysates with GFP-ERdd-pRS416 grown with different estradiol concentrations, and with GFP-pRS416 and empty pRS416 as controls. GFP-ERdd fusion protein abundance shows a clear dose response to estradiol. The experiment was performed once using on a single membrane, which was first developed with the anti-GFP antibody, stripped, and developed with the anti-GAPDH antibody.

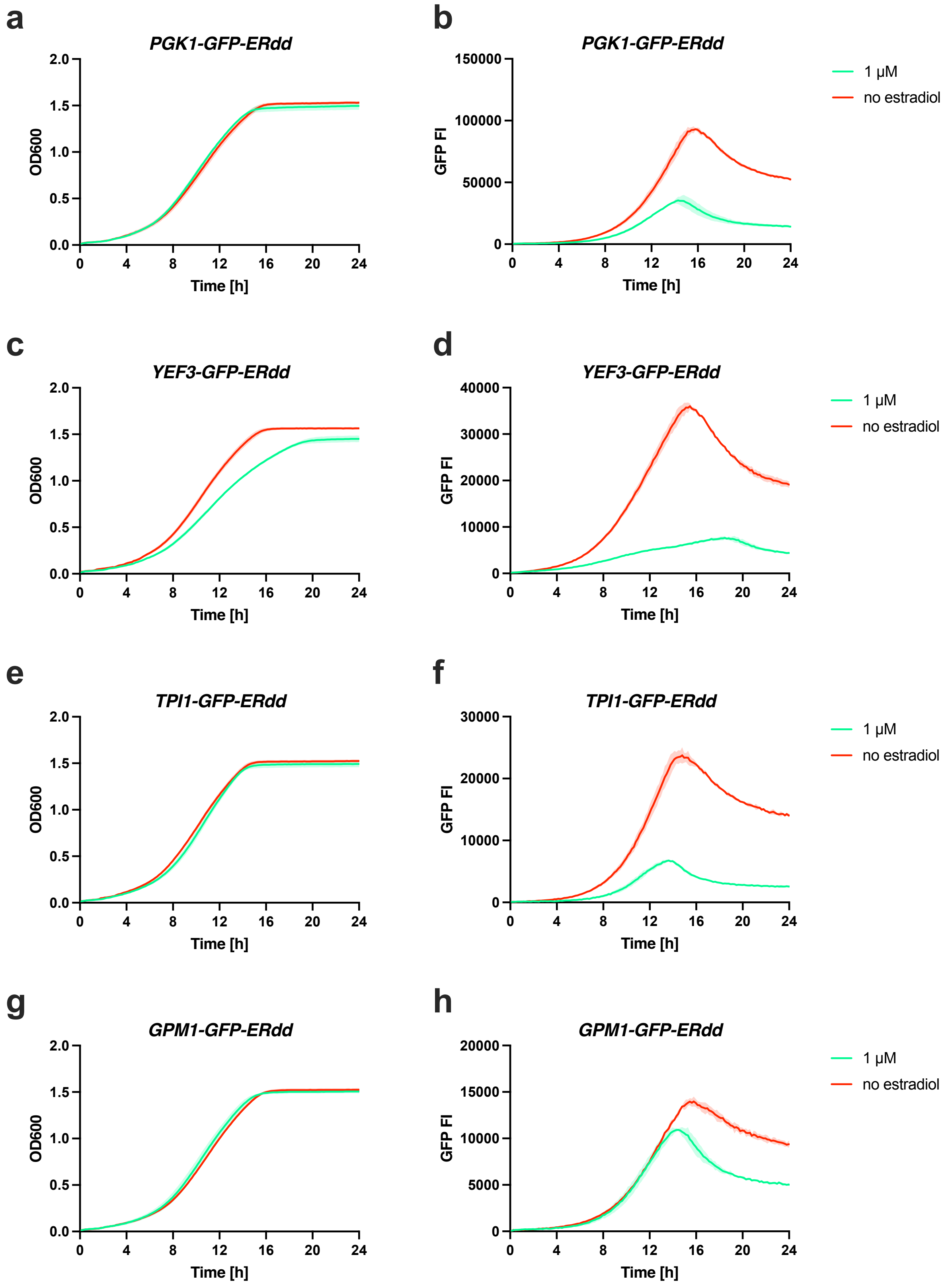

Figure S2: Liquid growth assays with recording of GFP fluorescence intensity of strains with GFP-ERdd fusions of four highly expressed essential genes. Optical density at 600 nm (**a**, **c**, **e**, **g**) to monitor growth and GFP fluorescence intensity (**b**, **d**, **f**, **h**) as measure for relative abundance of the respective essential GFP-ERdd fusion protein were recorded. Despite substantial reduction of essential fusion protein levels without estradiol was observed for each fusion protein, the strains showed no or only minor estradiol dependence. Shown are means with standard deviations from three replicates. Source data are provided as a Source Data file.

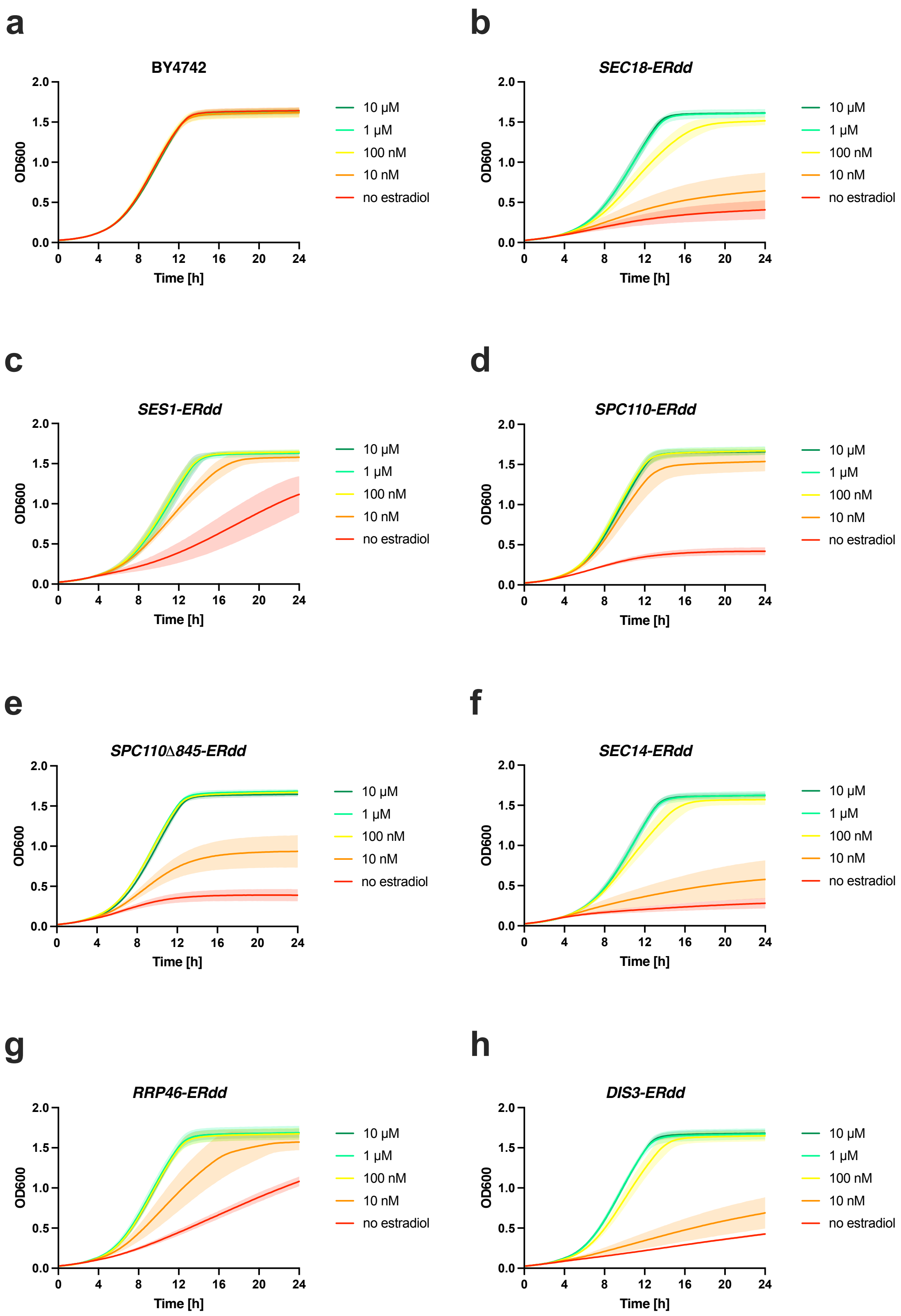

Figure S3: Liquid growth assays to assay estradiol dependence in singly ERdd-tagged strains. Strains with direct ERdd fusions of the six winner genes from the screening of GFP-ERdd fusions and the escape frequency-improved strain *SPC110Δ845-ERdd* were assayed. **a** is the parental strain BY4742. In the other panels strains with the following ERdd-tagged genes are shown: **b** *SEC18*, **c** *SES1*, **d** *SPC110*, **e** *SPC110Δ845*, **f** *SEC14*, **g** *RRP46*, and **h** *DIS3*. All seven ERdd strains show strong estradiol growth dependence but differ in response profiles. Notably, growth of *SPC110-ERdd*, *RRP46-ERdd* and *SPC110Δ845-ERdd* is fully restored at 100 nM estradiol. Shown are means with standard deviations from three independent experimental repeats. Source data are provided as a Source Data file.

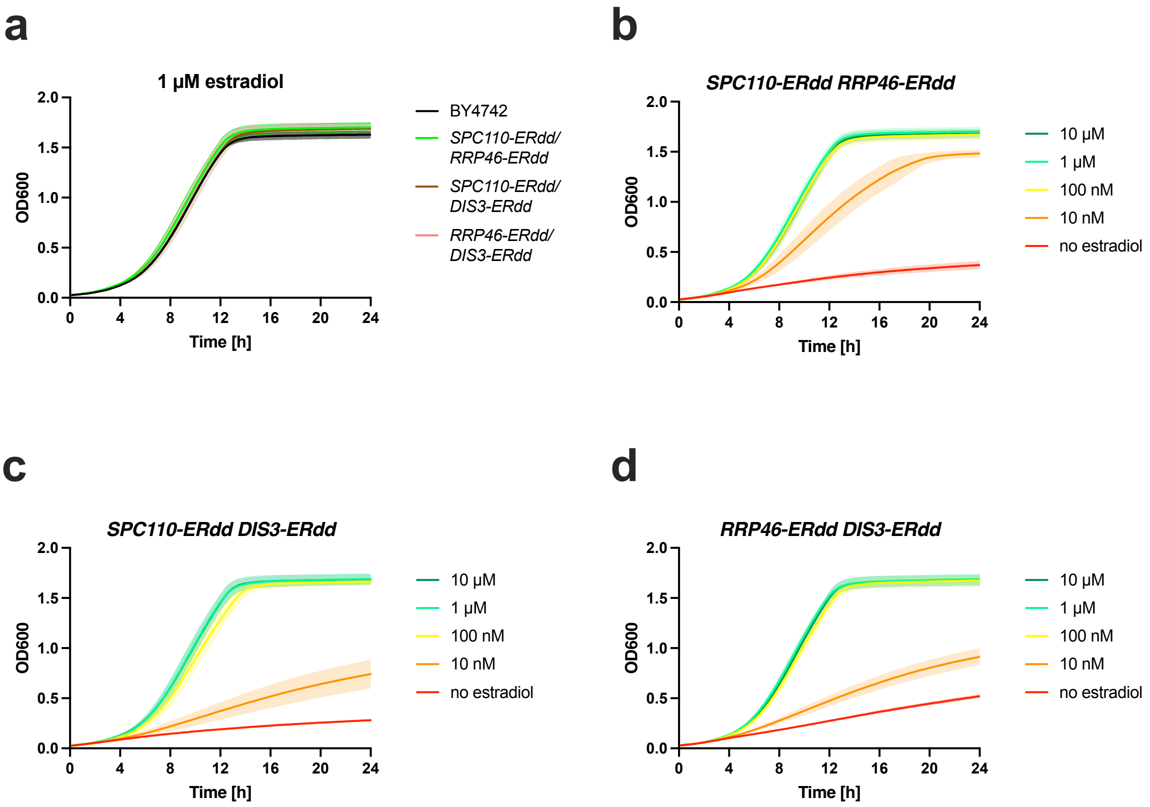

Figure S4: Liquid growth assays to assay estradiol dependence in strains with two select ERdd-tagged essential genes. **a** Growth of the three strains with two ERdd-tagged genes is indistinguishable from that of the parental strain BY4742. **b** shows growth curves of *SPC110-ERdd;RRP46-ERdd*, **c** of *SPC110-ERdd;DIS3-ERdd*, and **d** of *RRP46-ERdd;DIS3-ERdd*. Each graphs shows means with standard deviations from three independent experimental repeats. Source data are provided as a Source Data file.

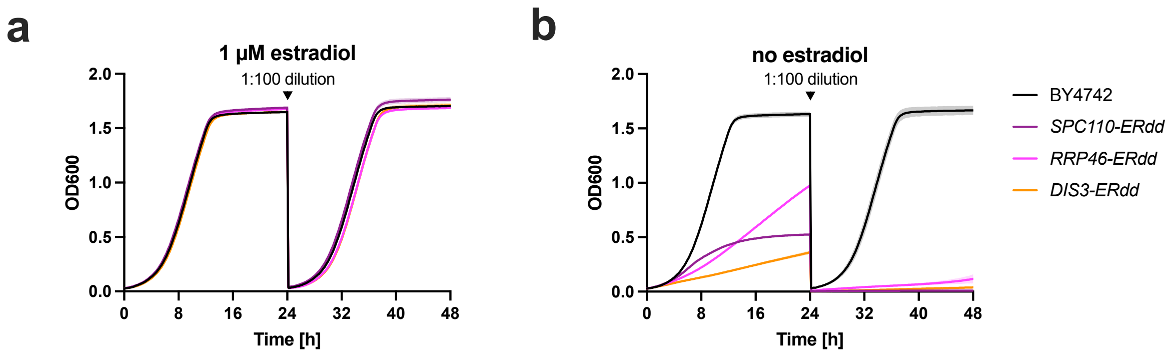

Figure S5: Sequential liquid growth assays of single ERdd strains with and without estradiol. **a** Shows respective growth curves with and **b** without estradiol. After a primary assay, cultures were back diluted 1:100 in the same medium, performing a secondary assay. Shown are means with standard deviations from five replicates each. Source data are provided as a Source Data file.

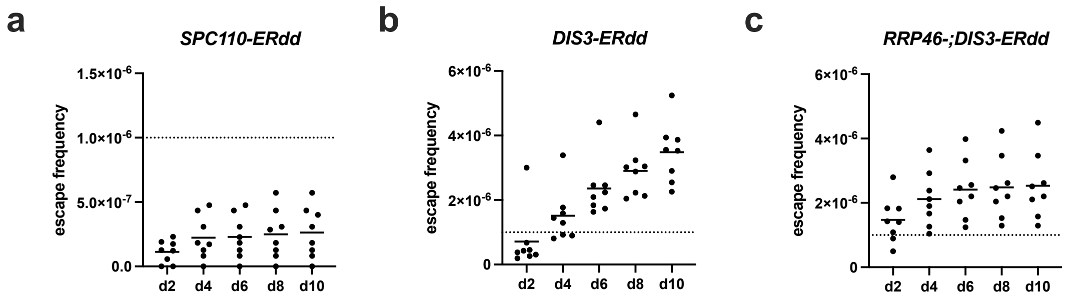

Figure S6: Standard escape assays over 10 days. Standard escape assays were carried out for **a** *SPC110-ERdd*, **b** *DIS3-ERdd* and **c** *RRP46-ERdd; DIS3-ERdd*, each with 8 biological replicates. Shown are individual escape frequencies as dots and means as dashes. In all graphs, an escape frequency of 1×10^{-6} is marked by a horizontal dashed line for ease of quantitative comparison between the graphs. Source data are provided as a Source Data file.

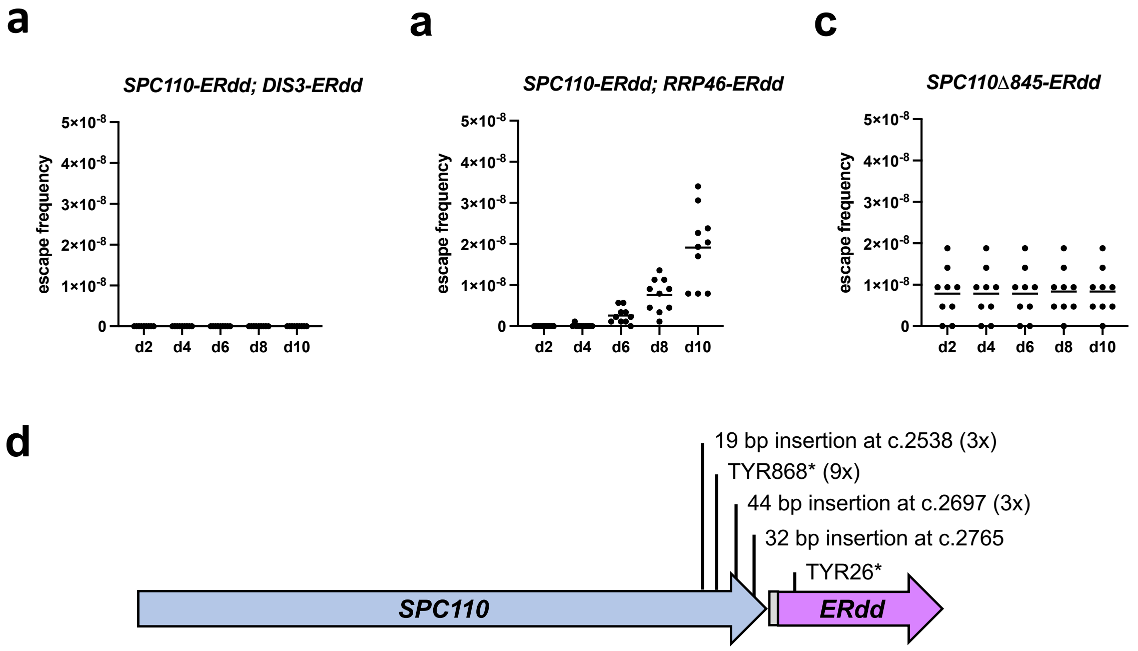

Figure S7: Low-frequency escape assays over 10 days after replica plating. Low-frequency escape assays were carried out for **a** *SPC110-ERdd/DIS3-ERdd,* **b** *SPC110-ERdd/RRP46-ERdd* and **c** *SPC110Δ845-ERdd*. Shown are individual values (9 technical replicates for *SPC110Δ845-ERdd* and 10 for the other two strains) as dots and means as dashes. Source data are provided as a Source Data file. **d** Escape mutations of *SPC110-ERdd*; *RRP46-ERdd* in the *SPC110-ERdd* fusion gene. All 18 sequenced escapees carried an escape mutation in *SPC110-ERdd*. Only five different escape mutations were found.

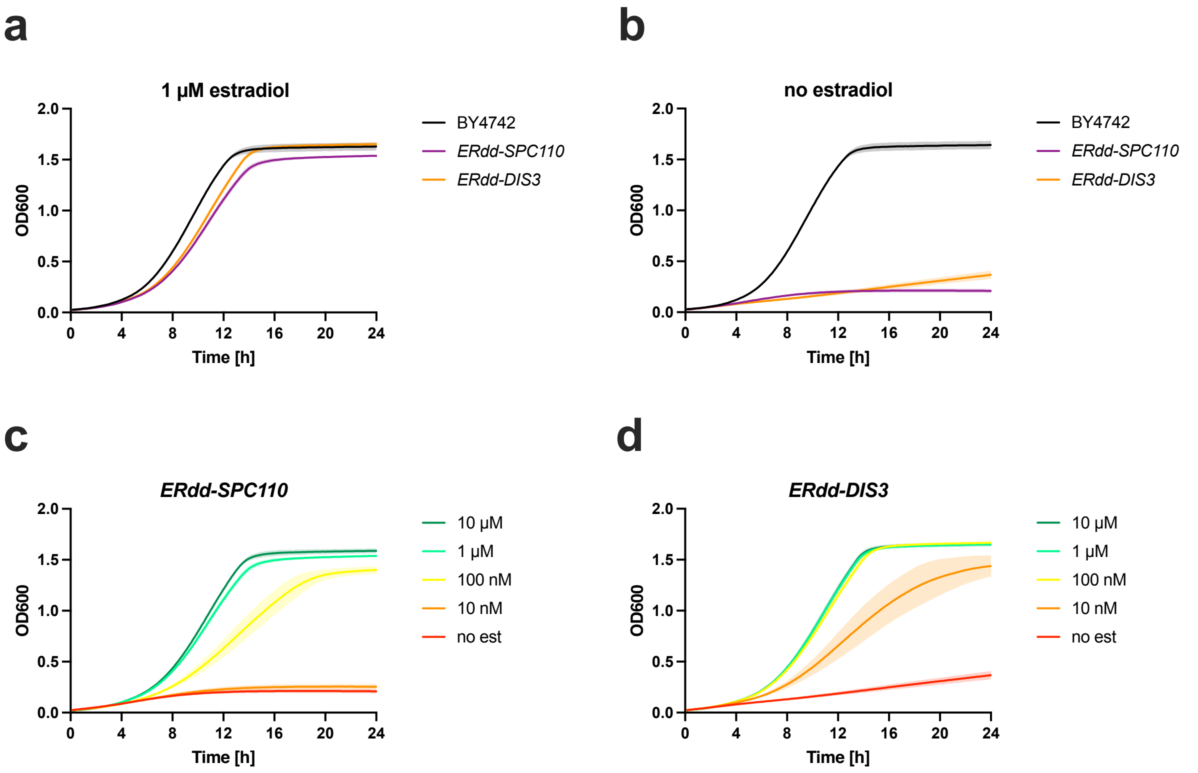

Figure S8: Estradiol dependence assays of N-terminal ERdd fusion strains. Growth is not fully restored for the N-terminal fusion strains at 1 µM estradiol. **b** Both N-terminal ERdd strains have a severe growth defect in the absence of estradiol. **c** Shows growth at different estradiol concentrations for *ERdd-SPC110*, and **d** for *ERdd-DIS3*. Each graphs shows means with standard deviations from three independent experimental repeats. Source data are provided as a Source Data file.

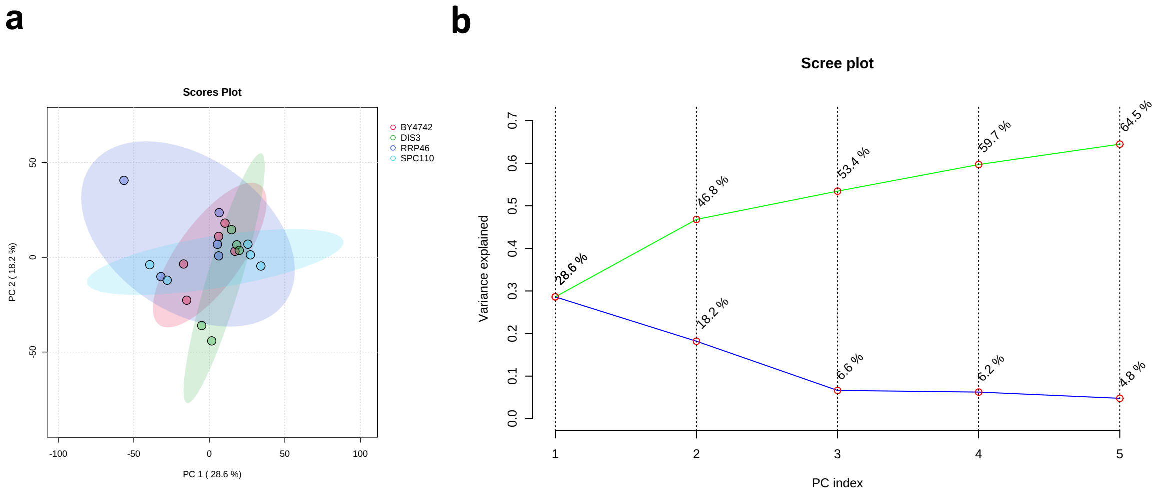

Figure S9: Principal component analysis of normalized peak intensities from metabolomic profiling of ERdd strains. a Scores plot between the principal components 1 and 2. None of the assessed ERdd strains (*DIS3-ERdd*, *RRP46-ERdd*, *SPC110-ERdd*) form a separable cluster, indicating the metabolomic profiles are indistinguishable from that of the parental strain BY4742. **b** Scree plot of principal component analysis. Source data are provided as a Source Data file.

Table S1: Generated strains with essential genes directly fused to ERdd.

Strain ID	ERdd-tagged gene(s)	Parent	Genotype
SHy110	*SEC18*	BY4742	*MATα his3Δ1 leu2Δ lys2Δ ura3Δ SEC18-ERdd*
SHy111	*SES1*	BY4742	*MATα his3Δ1 leu2Δ lys2Δ ura3Δ SES1-ERdd*
SHy112	*SPC110*	BY4742	*MATα his3Δ1 leu2Δ lys2Δ ura3Δ SPC110-ERdd*
SHy113	*SEC14*	BY4742	*MATα his3Δ1 leu2Δ lys2Δ ura3Δ SEC14-ERdd*
SHy114	*RRP46*	BY4742	*MATα his3Δ1 leu2Δ lys2Δ ura3Δ RRP46-ERdd*
SHy115	*DIS3*	BY4742	*MATα his3Δ1 leu2Δ lys2Δ ura3Δ DIS3-ERdd*
SHy117	*SPC110, RRP46*	SHy114	*MATα his3Δ1 leu2Δ lys2Δ ura3Δ SPC110-ERdd RRP46-ERdd*
SHy118	*SPC110, DIS3*	SHy112	*MATα his3Δ1 leu2Δ lys2Δ ura3Δ SPC110-ERdd DIS3-ERdd*
SHy119	*RRP46, DIS3*	SHy115	*MATα his3Δ1 leu2Δ lys2Δ ura3Δ RRP46-ERdd DIS3-ERdd*
SHy121	*SPC110Δ845*	BY4742	*MATα his3Δ1 leu2Δ lys2Δ ura3Δ SPC110Δ845-ERdd*
SHy166	*SPC110*	BY4742	*MATα his3Δ1 leu2Δ lys2Δ ura3Δ ERdd-SPC110*
SHy167	*DIS3*	BY4742	*MATα his3Δ1 leu2Δ lys2Δ ura3Δ ERdd-DIS3*

Table S2: Oligonucleotides used in this study to generate CRISPR guide constructs and confirm edits.

Oligonucleotide description	Sequence
HIS3MX6 guide sense	gactttTTCCCAAGCTACTCAGTCAC
HIS3MX6 guide anti-sense	aaacGTGACTGAGTAGCTTGGGAAaa
YBR080C guide sense	gactttTTGAAATAATTATGCGGATT
YBR080C guide anti-sense	aaacAATCCGCATAATTATTTCAAaa
YDR023W guide sense	gactttCTTTTTCTTGTCTTTACTAG
YDR023W guide anti-sense	aaacCTAGTAAAGACAAGAAAAAGaa
YDR356W guide sense	gactttGTAGCAGCGGGCGTATATCT
YDR356W guide anti-sense	aaacAGATATACGCCCGCTGCTACaa
YGR095C guide sense	gactttAATGGCTATACAACTAGACG
YGR095C guide anti-sense	aaacCGTCTAGTTGTATAGCCATTaa
YMR079W guide sense	gactttATAATATCATTTCATCGAAA
YMR079W guide anti-sense	aaacTTTCGATGAAATGATATTATaa
YOL021C guide sense	gactttAGAATTATTGTTAAAATAGA
YOL021C guide anti-sense	aaacTCTATTTTAACAATAATTCTaa
YDR356W 5' guide sense	gactttAAAGGGCTGATAAACACTCA
YDR356W 5' guide anti-sense	aaacTGAGTGTTTATCAGCCCTTTaa
YGR095C 5’ guide sense	gactttGATATTTTATGCATCTTAAC
YGR095C 5’ guide anti-sense	aaacGTTAAGATGCATAAAATATCaa
YOL021C 5’ guide sense	gactttCGGGAACTGACATGTTGTTT
YOL021C 5’ guide anti-sense	aaacAAACAACATGTCAGTTCCCGaa
YBR080C fw 2178	CAATGTCGGTATTAAAAAGACC
YBR080C rv 200ds	ATCATCTTTACCCGGTGG
YDR023W fw 1290	TTTGAGGAAATACATTCCAGGTG
YDR023W rv 220ds	GGGTTATGAGGGAACTATTTG
YDR356W fw 2736	GAGGATGAAAAGGATTGCTTTTTAC
YDR356W rv 230ds	AGATATACCCTACGTATGTTTTATGC
YGR095C fw 672	GTTCAGTTTACTGGAGCTG
YGR095C rv 250ds	AAAGCAAGCTGGATAACAC
YMR079W fw 594	ATCCAAGGGTGGGTTATAC
YMR079W rv 200ds	ATAATCTAATAGCTGAGTGGAAG
YOL021C fw 2907	ACCGAGGGATGTTTACG
YOL021C rv 230ds	ACGTTGCCATCGTTG
YDR356W 5’ fw	CGCGGATATCTTAAGCAACTTC
YDR356W 5’ rv	CATTATTGGCATTTGGAACCTT
YGR095C 5’ fw	TAAAGGTGACAAAATAGCGCCT
YGR095C 5’ rv	TATCCCTATTTCTGCTTGGACG
YOL021C 5’ fw	GATACATTGTGAGGGACCCATT
YOL021C 5’ rv	ATGAACTTCGGCAACTCATTTT
