# Supplementary material for: Engineering stringent genetic biocontainment of yeast with a protein stability switch
Source: Nat Commun. 2024 Feb 5;15:1060. doi: 10.1038/s41467-024-44988-8 (PMC10844650; doi:10.1038/s41467-024-44988-8)
Supplement: Supplementary file 2 — Reporting Summary [file 41467_2024_44988_MOESM2_ESM.pdf]

nature portfolio

Corresponding author(s): Yizhi Cai

Last updated by author(s): Jan 9, 2024

Reporting Summary

Nature Portfolio wishes to improve the reproducibility of the work that we publish. This form provides structure for consistency and transparency in reporting. For further information on Nature Portfolio policies, see our Editorial Policies and the Editorial Policy Checklist.

Statistics

For all statistical analyses, confirm that the following items are present in the figure legend, table legend, main text, or Methods section.

n/a	Confirmed	
☐	☒	The exact sample size (*n*) for each experimental group/condition, given as a discrete number and unit of measurement
☐	☒	A statement on whether measurements were taken from distinct samples or whether the same sample was measured repeatedly
☐	☒	The statistical test(s) used AND whether they are one- or two-sided<br>*Only common tests should be described solely by name; describe more complex techniques in the Methods section.*
☒	☐	A description of all covariates tested
☐	☒	A description of any assumptions or corrections, such as tests of normality and adjustment for multiple comparisons
☐	☒	A full description of the statistical parameters including central tendency (e.g. means) or other basic estimates (e.g. regression coefficient) AND variation (e.g. standard deviation) or associated estimates of uncertainty (e.g. confidence intervals)
☐	☒	For null hypothesis testing, the test statistic (e.g. *F*, *t*, *r*) with confidence intervals, effect sizes, degrees of freedom and *P* value noted<br>*Give P values as exact values whenever suitable.*
☒	☐	For Bayesian analysis, information on the choice of priors and Markov chain Monte Carlo settings
☒	☐	For hierarchical and complex designs, identification of the appropriate level for tests and full reporting of outcomes
☒	☐	Estimates of effect sizes (e.g. Cohen's *d*, Pearson's *r*), indicating how they were calculated

Our web collection on statistics for biologists contains articles on many of the points above.

Software and code

Policy information about availability of computer code

Data collection	Acquisition of optical densities and fluorescence on a BioTek Synergy H1 was done using the Agilent software Gen5 v3.03. For collection of MS data on the Exploris 480, ThermoFisher Scientific software Xcalibur v4.4.16.14 was used. The Q Exactive Plus was run with its dedicated ThermoFisher software 2.11 Build 3005. The NovoSeq Control Software v1.8 was used for NGS data acquisition on an Illumina NovaSeq 6000.
Data analysis	The following tools were used by Eurofins for analysis of Illumina sequencing data (Variant Analysis Pipeline v2.4.3), including sequence cleaning, mapping and alignment, variant discovery and annotation, and QC and coverage analysis: fastp v0.200, BWA v0.7.17, Sentieon v202112.02, BCFtools v1.15, samtools v1.10, sambamba v0.6.8, RTG_Tools v3.12.1, BCFtools v1.15, VCFtools v0.1.16, Perl v5.28.2, R v4.1.3. Based on the outputted variant calling files, we annotated variants using VariantAnnotation v1.40.<br><br>Analysis of proteome MS spectra was performed with Proteome Discoverer v2.5.0.400. Metabolomics data analysis was performed using MetaboAnalyst 5.0.

For manuscripts utilizing custom algorithms or software that are central to the research but not yet described in published literature, software must be made available to editors and reviewers. We strongly encourage code deposition in a community repository (e.g. GitHub). See the Nature Portfolio guidelines for submitting code & software for further information.

Data

Policy information about availability of data

All manuscripts must include a data availability statement. This statement should provide the following information, where applicable:

- Accession codes, unique identifiers, or web links for publicly available datasets
- A description of any restrictions on data availability
- For clinical datasets or third party data, please ensure that the statement adheres to our policy

Whole genome sequencing data has been deposited at SRA with the BioProject number PRJNA923142. Proteome data is available via ProteomeXchange with identifier PXD042326.
The Toronto BY4742 reference (available from https://downloads.yeastgenome.org/sequence/strains/BY4742/BY4742_Toronto_2012) was used for mapping, variant calling and annotation. For analysis of protein MS spectra in Proteome Discoverer, NCBI txid559292 v2022-04-30 (Saccharomyces cerevisiae S288C) was used.

Research involving human participants, their data, or biological material

Policy information about studies with human participants or human data. See also policy information about sex, gender (identity/presentation), and sexual orientation and race, ethnicity and racism.

Reporting on sex and gender	n/a
Reporting on race, ethnicity, or other socially relevant groupings	n/a
Population characteristics	n/a
Recruitment	n/a
Ethics oversight	n/a

Note that full information on the approval of the study protocol must also be provided in the manuscript.

Field-specific reporting

Please select the one below that is the best fit for your research. If you are not sure, read the appropriate sections before making your selection.

☒ Life sciences ☐ Behavioural & social sciences ☐ Ecological, evolutionary & environmental sciences

For a reference copy of the document with all sections, see nature.com/documents/nr-reporting-summary-flat.pdf

Life sciences study design

All studies must disclose on these points even when the disclosure is negative.

Sample size	No statistic sample size calculation was performed, and no prior assumptions about effect sizes were made.<br>The primary screening for estradiol-dependent growth of the created GFP-ERdd library (775 strains) was done on semisolid media with a single experimental repeat. The more focused secondary screening of 46 of those strains was performed in three independent experiments in liquid culture, with a high degree of reproducibility for most strains. The 6 highest scoring strains in the secondary screening are among the 15 highest scoring strains of the primary screen, indicating sample sizes were adequate to identify the best performers in the library.
Data exclusions	For analysis of shotgun proteomics data, only proteins identified by at least two unique peptides were included. This pre-established criterion was chosen to ensure high confidence in the protein identity of analyzed abundances. No other data were excluded.
Replication	Growth assays were performed in at least three independent experiments each to ensure reproducibility. All attempts at replication were successful.
Randomization	No arbitrary experimental group allocation took place in this study, hence randomization procedures do not apply.
Blinding	No arbitrary experimental group allocation took place in this study, hence blinding procedures do not apply.

Reporting for specific materials, systems and methods

We require information from authors about some types of materials, experimental systems and methods used in many studies. Here, indicate whether each material, system or method listed is relevant to your study. If you are not sure if a list item applies to your research, read the appropriate section before selecting a response.

Materials & experimental systems

n/a	Involved in the study
☐	☒ Antibodies
☒	☐ Eukaryotic cell lines
☒	☐ Palaeontology and archaeology
☒	☐ Animals and other organisms
☒	☐ Clinical data
☒	☐ Dual use research of concern
☒	☐ Plants

Methods

n/a	Involved in the study
☒	☐ ChIP-seq
☒	☐ Flow cytometry
☒	☐ MRI-based neuroimaging

Antibodies

Antibodies used

anti-GFP GF28R, Thermo Fisher, Catalog Number 14-6674-82, LOT 2525217
anti-GAPDH GA1R, antibodies.com, Catalog Number A85271 (identical to Abcam ab125247), LOT 31087
rabbit anti-mouse IgG antibody, Sigma Aldrich, Catalog Number A9044-2ml, LOT unknown

Validation

Data for the anti-GFP antibody is available here:
https://www.thermofisher.com/antibody/product/GFP-Antibody-clone-GF28R-Monoclonal/14-6674-82
It contains western blot validation data of the antibody used at 1 µg/ml with HEK cell lysates. Further, it links to 8 publications, in which this antibody product has been used in western blot to detect GFP used as a heterelogous protein tag. In the present manuscript, for western blotting of yeast lysates with GFP-ERdd, a GFP only and a no GFP control were included (see Figure S1).

Data for the anti-GAPDH antibody is available here:
https://www.antibodies.com/gapdh-antibody-ga1r-a85271
According to the provided information, the antibody is suitable for western blot and reactive with endogenous GAPDH of S. cerevisiae. It links to 16 publications in which it has been used, at least one of which uses it in western blot of S. cerevisiae lysates (doi: 10.1038/srep32117).

Plants

Seed stocks

Report on the source of all seed stocks or other plant material used. If applicable, state the seed stock centre and catalogue number. If plant specimens were collected from the field, describe the collection location, date and sampling procedures.

Novel plant genotypes

Describe the methods by which all novel plant genotypes were produced. This includes those generated by transgenic approaches, gene editing, chemical/radiation-based mutagenesis and hybridization. For transgenic lines, describe the transformation method, the number of independent lines analyzed and the generation upon which experiments were performed. For gene-edited lines, describe the editor used, the endogenous sequence targeted for editing, the targeting guide RNA sequence (if applicable) and how the editor was applied.

Authentication

Describe any authentication procedures for each seed stock used or novel genotype generated. Describe any experiments used to assess the effect of a mutation and, where applicable, how potential secondary effects (e.g. second site T-DNA insertions, mosiacism, off-target gene editing) were examined.
